# Acute Necrotizing Encephalopathy of Childhood; A Case Report

**Published:** 2013

**Authors:** Mohammad Reza SALEHIOMRAN, Hajighorban NOOREDDINI, Fatemeh BAGHDADI

**Affiliations:** 1 Associate Professor, Non-Communicable Pediatric Diseases Research Center, Babol University of Medical Sciences, Mazandaran, Iran; 2 Pediatric Radiologist, Amirkola Children Hospital, Babol University of Medical Sciences, Mazandaran, Iran; 3 Pediatrician, Babol University of Medical Sciences, Mazandaran, Iran

**Keywords:** Acute Necrotizing Encephalopathy, Epilepsy, Pediatrics

## Abstract

Acute necrotizing encephalopathy of childhood (ANEC) is a disease characterized by respiratory or gastrointestinal infection and high fever accompanying with rapid alteration of consciousness and seizures. This disease is nearly exclusively seen in East Asian infants and children who had previously been completely healthy. Serial magnetic resonance imaging examinations have demonstrated symmetric lesions involving the thalami, brainstem, cerebellum, and white matter in this disease. The condition accompanies a poor prognosis with high morbidity and mortality rates. A 22-month-old toddler with ANEC hospitalized in Amirkola Children Hospital is being reviewed.

## Introduction

Acute necrotizing encephalopathy of childhood (ANEC) is an atypical encephalopathy seen almost exclusively in previously healthy young children or infants of East Asian including Japan and Taiwan (1). However, its sporadic cases have been reported from all around the world (2,3).

The real etiology and pathogens of ANEC remains nebulous; however, mycoplasma, influenza virus, herpes simplex virus, and human herpes virus-6 are among the most common infections that intensify the disease (4-6). It is believed that ANEC is similar to metabolic and immune-mediated reactions. Cytokines such as tumor necrotizing factor-α and interleukins 1 and 6 can speed up the disease (6).

Disease initiates sonorously accompanying with seizures, rapid neurologic decline, vomiting, and different grades of liver disorder (3). No specific treatment or preventive method has been determined for this disease and a poor prognosis with less than 10% of complete recovery is generally expected in the patients. Most of them experience rapid neurologic decline and death. The intensity of involvement and magnetic resonance imaging (MRI) lesions are clearly related with the outcome (7). We present a 22-month-old girl with ANEC who was admitted to Amirkola Children Hospital to know more about this atypical disease.

## Case Report

A previously healthy, 12-kg, 22-month-old female toddler was referred to us with high fever, non-bloody and non-xanthic jumpy vomiting, and non-bloody watery diarrhea following a trip. Her height was 80 cm (below the percentile 25%) and head circumference was 47 cm (percentile 50%). Several hours later, her level of consciousness decreased (Glasgow coma scale=7), she developed generalized tonic-clonic seizures, and was transferred to PICU. Quadripelagic spasms with greater intensity in the right upper and lower limbs as well as spasity and severe dystonia were then noted. In the course of the disease, she was transferred to Amirkola Children Hospital in Babol. On the first examination after admission to this center, her complete blood count, PT, PTT, and urine analysis were normal. C reactive protein, erythrocyte sedimentation rate, blood sugar, blood urea nitrogen, creatinine, calcium, sodium, potassium, aspartate transaminase, alanine transaminase, alkaline phosphatase, total bilirubin, and direct bilirubin were reported to be 2 mg/dL, 7 mm/hr, 94 mg/dL, 9.2 mg/dL, 0.6 mg/dL, 9.4 mg/dL, 140 mEq/L, 5.2 mEq/L, 100 U/L, 119 U/L, 247U/L, 1.1 mg/dL, and 0.5 mg/dL, respectively. Evaluation of the cerebrospinal fluid (CSF), blood ammonia, lactate, and pyruvate were also normal. Urine reducing substances, amino acids chromatography and blood gas were normal. Anticonvulsant therapy with phenytoin, Phenobarbital, and midazolam drip was begun. Respiratory support by mechanical ventilation was also initiated. Acyclovir (Zovirax), dexamethasone, intravenous immunoglobulin (IVIG), amikacin (for UTI), and imipenem were prescribed. 

**Fig 1:**
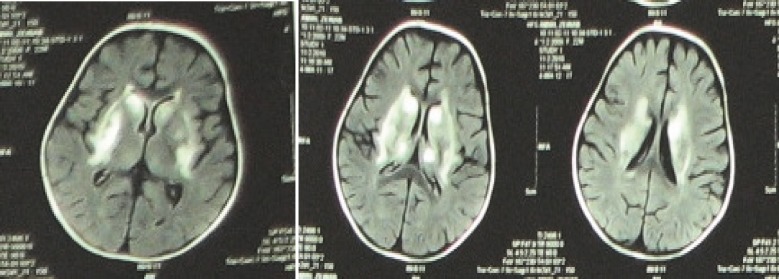
Symmetric hyperintensities in caudate nucleus, putamen, globus pallidus, the thalamus on both sides

Diazepam (0.2 mgr /kgr q6h), levodopa(10mgr/day), and trihexifenidilo(2mgr/6h) were used to treat dystonia. Although dystonia and spasity gradually declined, mental and physical sequelae developed. She therefore became a candidate for percutaneous endoscopic gastrostomy (PEG). MRI revealed increased T2 signal intensity in the thalami, basal ganglia (putamen and globus pallidus), and periventricular white matter presenting hemorrhage accompanied with necrosis in those regions (Fig.1). 

The patient underwent supportive care including physiotherapy, speech therapy, and outpatient supports.

## Discussion

Acute necrotizing encephalopathy of childhood is a disease entity seen in young previously healthy children (2,3). Our case was a previously healthy child whose disease initiated with high fever, seizures, and fast neurologic disturbances (8).

No clear relation has been determined between the occurrence of this disease in special geographical regions and its genetics in Asian race. San Millan has reported several cases of this disease in Spain (9). It initiates after a fever malady accompanied by consciousness decline, seizures, spasms, and neurologic disorders (10).

Although the etiology remains nebulous, influenza A and B are the most commonly infections associated with the disease (11). A 10-year-old child with ANEC has been described who had influenza A confirmed by serology two weeks after the disease had started. Although MRI revealed increased T2 signal intensity in the thalami, he finally improved completely. He had been admitted to the hospital in influenza A epidemics (12); in our study no virus examination was done. In our patient, the disease followed a gastrointestinal infection. In Skelton and associates’ study, a case of ANEC was reported after fever and diarrhea, as well (6). 

Start of the disease is terribly sonorous with neurologic symptoms as in our case. Kurachi and colleagues reported a 17-month-old male toddler with horizontal gaze palsy who was suffering from ANEC (13). 

Generally, no specific changes are seen in the laboratory tests of these patients, except for a rise in CSF protein without pleocytosis. Liver enzymes rise and liver disorders are seen in some patients (6), as seen in our patient. 

**Fig 2:**
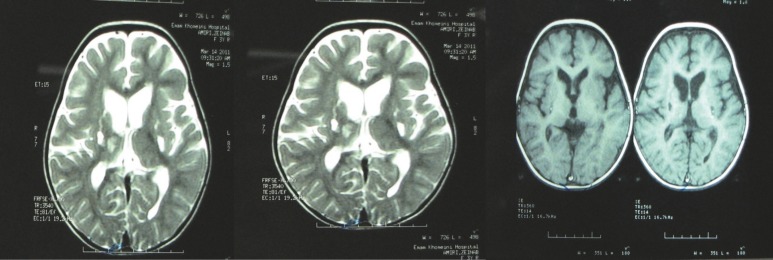
Bilateral focal areas of hyperintensites in the thalamus and putamen symmetrically on both sides due to necrosis

MRI revealed increased T2 signal intensity in the thalami, supratentorial white and grey matter, and bilateral cerebellar hemispheres (10). These signs were also seen in our patient. The patient’s After 4 months, MRI showed lateral ventricular dilatation accompanied with high-signal intensity in thalami and basal ganglia in T2 and low-signal intensity in T1 and FLAIR view implicating necrosis and brain gliosis in those regions. Second MRI revealed lower intensity than the first MRI (Fig2).

According to the previous studies, no significant relation exists between the extent of the brain damage and disease outcome (7). This damage-mainly located in the brainstem- includes bleeding and cavitation. Some cases of full recovery have been reported; but, in our case the neurologic function decline occurred rapidly and then gradually improved. 

Weng and coworkers reported spinal cord involvement in a 2-year-old girl with ANEC (11). Our case, however, did not show any sign and symptom of spinal cord. These patients are usually treated by supportive care including hydration, electrolyte balancing, prevention of infection, and seizure and spasm control. Treatment with levodopa has also been suggested. We performed serum therapy, infection prevention, supportive ventilation, and administered anticonvulsants, L-dopa, trihexyphenidyl, and IVIG. Mortality and morbidity of these patients are high with less than 10% complete recovery (8). Most patients face with intense neurologic decline. Although the involvement region and neurologic lesions are broad, Wong and colleagues devised an MRI-based scoring system (7). Focal cystic degeneration and cortical atrophy were more seen on convalescent imaging of the patients who showed full clinical improvement. But, a poor prognosis was seen in children who had petechial hemorrhage and cavity lesions in their MRIs. In our patient, the limbs spasms decreased after 18 months of follow-up. She was gradually able to eat, drink, and walk with support and communicated non-verbally and verbally to some extent. However, she has major psychomotor sequelae and speech disturbances, yet.

**In conclusion, **although ANEC is a rare disease, it should not be underestimated.
